# Supplementary material for: Large-scale experimental evidence of carbon-mediated N and P co-amplification in proglacial soils
Source: Nat Commun. 2025 Jul 31;16:7028. doi: 10.1038/s41467-025-62425-2 (PMC12314028; doi:10.1038/s41467-025-62425-2)
Supplement: Supplementary file 1 — Supplementary Information [file 41467_2025_62425_MOESM1_ESM.pdf]

Supplementary_Information

This file includes:

1. Figure S1
2. Figure S2
3. Nitrification experiment and results
4. Figure S4
5. Table S1
6. Table S2
7. Source data

1. Figure S1

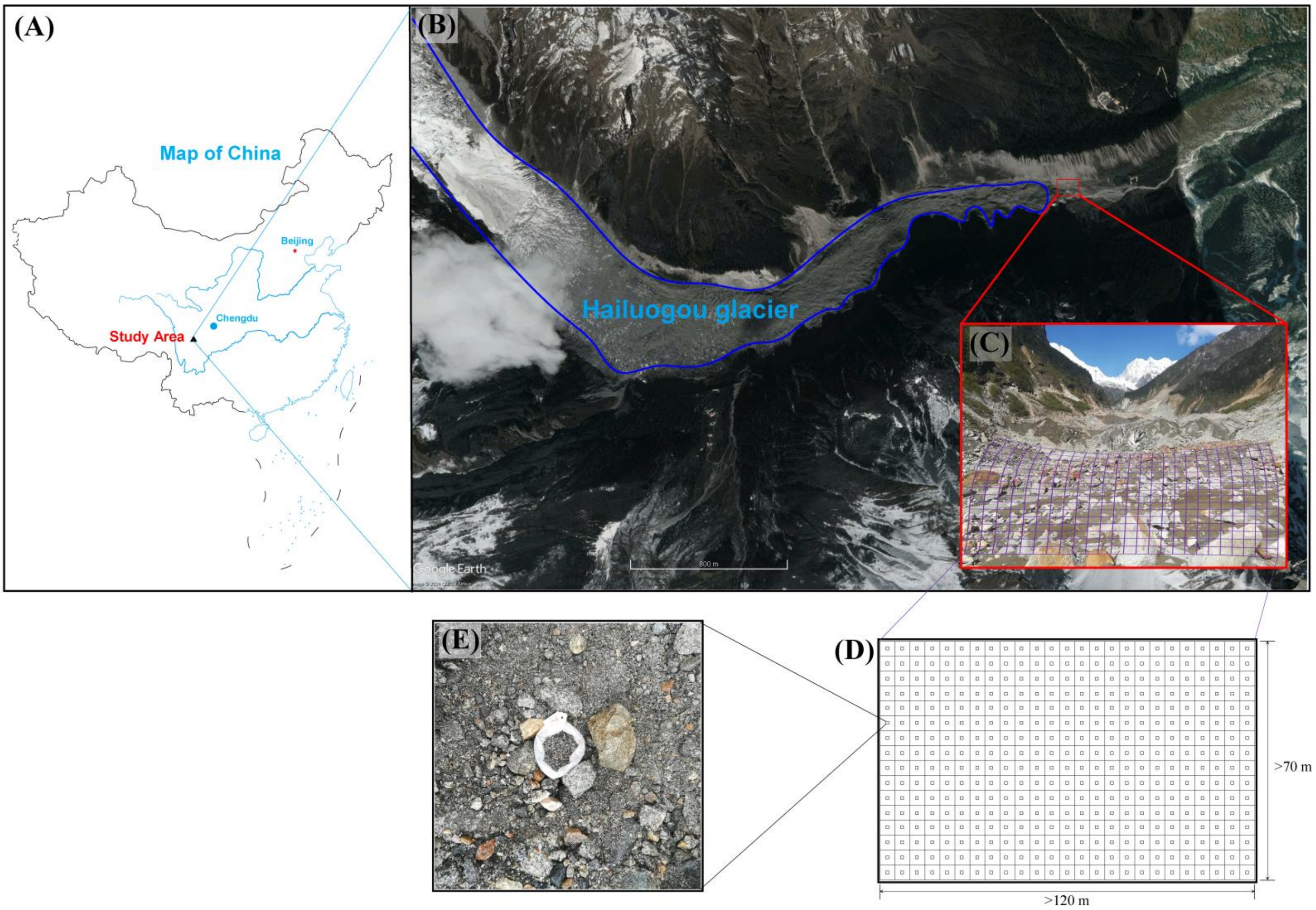

Fig. S1 | Geographical overview of the study area and soil column setup. **(A)** Map of China. **(B)** Hailuogou glacier foreland. The red box represents the most recently deglaciated region of the Hailuogou Glacier. **(C)** A real-scene photograph of the study area. **(D)** A schematic diagram of the layout of the plots. **(E)** Soil columns that were contained in tubular polyethylene bag and placed back in their original position

2. Figure S2

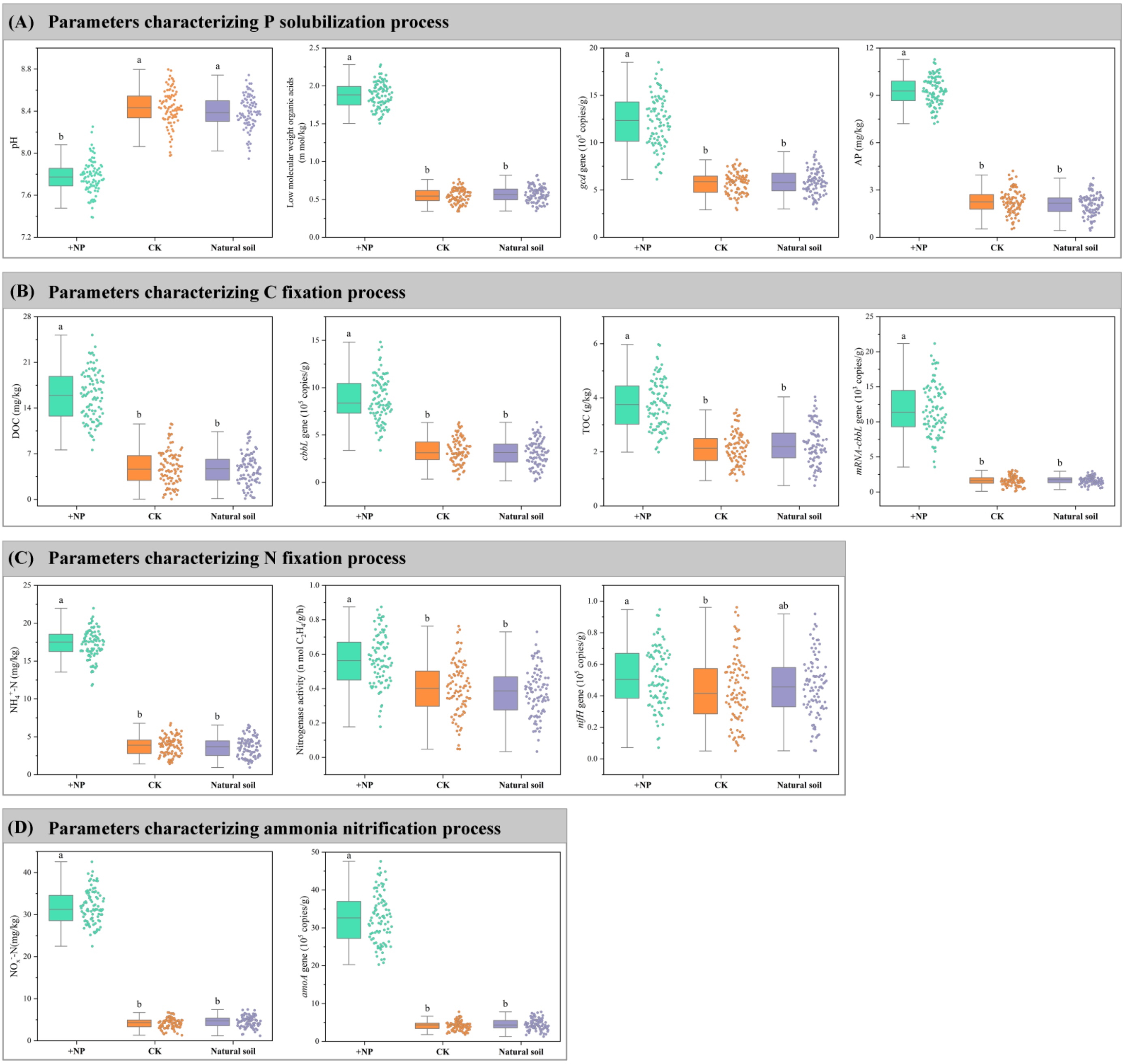

Fig. S2 | The effects of the treatment with combined N and P addition on soil processes in the early-stage soils over a three-year period (n= 90 plot replications per treatment). The band in the middle of each box represents the median, and top and bottom of the box represent the first and third quartiles, respectively. The error bars show 1.5 × interquartile range. Data values of indicators are indicated by the dots on the right side of the box. Different lowercase letters over the error bars indicate significant differences ($p < 0.05$) among different treatments. The '+NP ': the treatment with combined N and P addition; The 'CK': control treatments; 'Natural soil': soils that have not undergone any treatment. **(A)** Effects of the '+NP' treatment on parameters characterizing P solubilization process. **(B)** Effects of the '+NP' treatment on parameters characterizing C fixation process. **(C)** Effects of the '+NP' treatment on parameters characterizing N fixation process. **(D)** Effects of the '+NP' treatment on parameters characterizing ammonia nitrification process.

3. Nitrification experiment and results

1.1 Methods

To confirm that nitrification significantly contributed to the pH decrease in the ammonium addition groups, incubation experiments with NH_4Cl were conducted according to the following treatments.

To create conditions for adjusting the experimental soil to the average soil moisture content of the study area, the sieved soil was slightly air-dried.

Based on these air-dried soils, the experimental treatments (each with 6 replicates) were set up as follows:

N addition+ Non-Sterilization treatment (+N): 10 g of air-dried soil was placed into a 120 ml culture bottle, followed by the addition of 1 g of nitrogen (equivalent to 3.82 g of ammonium chloride). Sterile water was then added to adjust the moisture content to 9%, and the mixture was thoroughly homogenized.

Blank+ Non-Sterilization treatment (CK0): 10 g of air-dried soil was placed into a 120 ml culture bottle, and sterile water was added to adjust the moisture content to 9%. Then, 3.82 g of air-dried soils was added to the culture bottle and thoroughly mixed.

Blank + Sterilization treatment (CK1): 10 g of air-dried soil was placed into a 120 ml culture bottle, and sterile water was added to adjust the moisture content to 9%. Then, 3.82 g of air-dried soils was added to the culture bottle and thoroughly mixed. The soil was then sterilized using an autoclave.

N addition + Sterilization treatment (CK2): 10 g of air-dried soil was placed into a 120 ml culture bottle, and sterile water was added to adjust the moisture content to 9%. The soil was sterilized using an autoclave. Under a sterile workbench, 1 g of nitrogen (equivalent to 3.82 g of ammonium chloride) was added to the culture bottle and thoroughly mixed.

In order to ensure the breathability of the culture flasks and prevent the entry of bacteria, all the bottle mouths are wrapped with antibacterial masks. After all the culture flasks are cultivated in a dark environment at 20 ℃ for 90 days, soil pH is determined.

1.2 Results

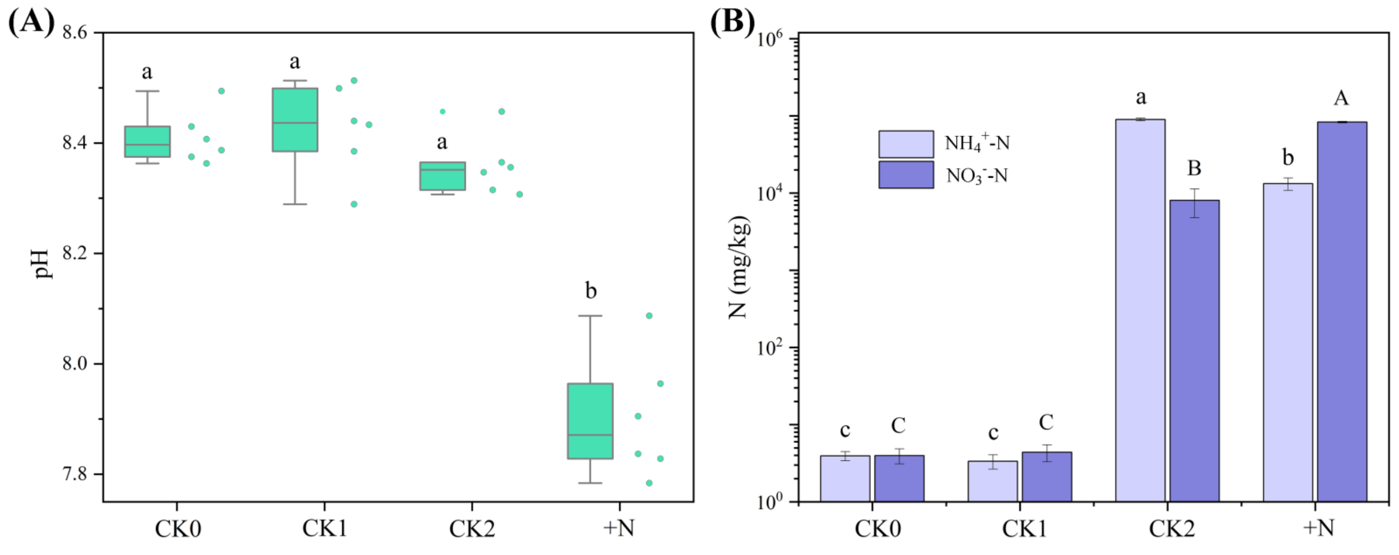

Fig. S3 | Under the conditions of the cultivation experiment, nitrogen addition reduces the soil pH through the process of nitrification (n=6). Figure **(A)**: Different lowercase letters indicate significant differences in pH between treatments at the statistical level of $p < 0.05$. Figure **(B)**: Different lowercase letters indicate significant differences in N-NH_4^+ between treatments at the statistical level of $p < 0.05$; Different uppercase letters indicate significant differences in N-NO_3^- between treatments at the statistical level of $p < 0.05$. CK0: Blank+ Non-Sterilization treatment; CK1: Blank + Sterilization treatment; CK2: N addition + Sterilization treatment; +N: N addition+ Non-Sterilization treatment.

4. Figure S4

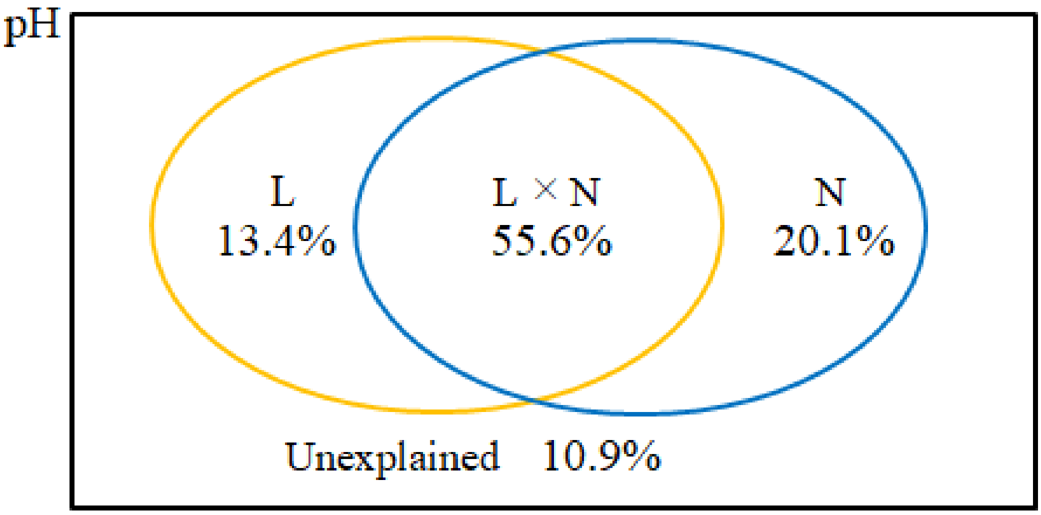

Fig. S4 | Quantifying the contributions of low molecular weight organic acids (LMWOA) and the ammonia - oxidation process (measured by NO_3^-–N, the product of the ammonia - oxidation process) to pH using partial Redundancy Analysis (partial RDA, n=90) based on the N addition treatment. In the figure, L represents LMWOA, and N represents NO_3^-–N.

5. Table S1

Table S1 | The design table of the experiments described in the manuscript (excluding the experiment of adding nitrogen and phosphorus simultaneously)

Treatment code	N addition amount ($g\ N\ m^{-2}\ year^{-1}$)	P addition amount ($g\ P\ m^{-2}\ year^{-1}$)	The number of replicates (sample size)	Experimental duration (year)
+N	10	0	90	3
+P	0	10	90	3
CK	0	0	90	3

6. Table S2

Table S2 | Primers and PCR conditions used for amplification of functional genes.

Primer name	Primer sequence (5'-3')	Amplified gene	PCR conditions	Reference
gcd-FW	CGGCGTCATCCGGGSITIYRAYRT	*gcd*	95 ℃/7 min	[1,2]
gcd-RW	GGGCATGTCCATGTCCCAIADRTCRTG		30 cycles of	
			95 ℃/1 min	
			60 ℃/1 min	
			72 ℃ /45 s	
cbbL-RedF	AAGGAYGACGAGAACATC	*cbbL*	95 ℃/6 min,	[3]
cbbL-RedR	TCGGTCGGSGTGTAGTTGAA		35 cycles of	
			95 ℃/1 min	
			57 ℃/2 min	
			72 ℃/2 min	
PolyF	TGCGAYCCSAARGCBGACTC	*nifH*	95 ℃/10min,	[4]
PolyR	ATSGCCATCATYTCRCCGGA		35 cycles of	
			95 ℃/30 s	
			62 ℃/30 s	
			72 ℃/45 s	
amoA-1F	GGGGTTTCTACTGGTGGT	*amoA*	95 ℃/3min,	[5]
amoA-2R	CCCCTCGGGAAAGCCTTCTTC		40 cycles of	
			95 ℃/20 s	
			56 ℃/30 s	
			72 ℃/30 s	

References:

1. Wang, F., Wei, X., Zhang, L. & Feng, G. Long-term fertilisation management changes bacterial phoD and gcd gene communities and abundances in the rhizosphere of cotton (Gossypium hirsutum L.) grown in a grey desert soil. Rhizosphere 28, 100797 (2023).
2. Bergkemper, F. et al. Novel oligonucleotide primers reveal a high diversity of microbes which drive phosphorous turnover in soil. Journal of Microbiological Methods 125, 91–97 (2016).
3. Yousuf, B., Keshri, J., Mishra, A. & Jha, B. Application of targeted metagenomics to explore abundance and diversity of CO2-fixing bacterial community using cbbL gene from the rhizosphere of Arachis hypogaea. Gene 506, 18–24 (2012).
4. Gaby, J. C. & Buckley, D. H. The Use of Degenerate Primers in qPCR Analysis of Functional Genes Can Cause Dramatic Quantification Bias as Revealed by Investigation of nifH Primer Performance. Microb Ecol 74, 701–708 (2017).
5. Xia, W., Bowatte, S., Jia, Z. & Newton, P. Offsetting N2O emissions through nitrifying CO2 fixation in grassland soil. Soil Biology and Biochemistry 165, 108528 (2022).

7. Source data

The source data generated in this study are provided in “Source Data file.xlsx”
