# Supplementary material for: Large-scale experimental evidence of carbon-mediated N and P co-amplification in proglacial soils
Source: Nat Commun. 2025 Jul 31;16:7028. doi: 10.1038/s41467-025-62425-2 (PMC12314028; doi:10.1038/s41467-025-62425-2)
Supplement: Supplementary file 3 — Reporting Summary [file 41467_2025_62425_MOESM3_ESM.pdf]

nature portfolio

Corresponding author(s): Hongyang Sun, Dong Yu

Last updated by author(s): Jul 19, 2025

Reporting Summary

Nature Portfolio wishes to improve the reproducibility of the work that we publish. This form provides structure for consistency and transparency in reporting. For further information on Nature Portfolio policies, see our Editorial Policies and the Editorial Policy Checklist.

Statistics

For all statistical analyses, confirm that the following items are present in the figure legend, table legend, main text, or Methods section.

n/a	Confirmed	
☐	☒	The exact sample size (*n*) for each experimental group/condition, given as a discrete number and unit of measurement
☐	☒	A statement on whether measurements were taken from distinct samples or whether the same sample was measured repeatedly
☐	☒	The statistical test(s) used AND whether they are one- or two-sided<br>*Only common tests should be described solely by name; describe more complex techniques in the Methods section.*
☐	☒	A description of all covariates tested
☐	☒	A description of any assumptions or corrections, such as tests of normality and adjustment for multiple comparisons
☐	☒	A full description of the statistical parameters including central tendency (e.g. means) or other basic estimates (e.g. regression coefficient) AND variation (e.g. standard deviation) or associated estimates of uncertainty (e.g. confidence intervals)
☐	☒	For null hypothesis testing, the test statistic (e.g. F, t, r) with confidence intervals, effect sizes, degrees of freedom and P value noted<br>*Give P values as exact values whenever suitable.*
☒	☐	For Bayesian analysis, information on the choice of priors and Markov chain Monte Carlo settings
☒	☐	For hierarchical and complex designs, identification of the appropriate level for tests and full reporting of outcomes
☒	☐	Estimates of effect sizes (e.g. Cohen's *d*, Pearson's *r*), indicating how they were calculated

Our web collection on statistics for biologists contains articles on many of the points above.

Software and code

Policy information about availability of computer code

Data collection	No software was used for data collection
Data analysis	One-way analysis of variance (ANOVA) and linear regression analyses were conducted using IBM SPSS Statistics 21 and Origin 2022, respectively. Structural equation modeling (SEM)was performed using AMOS 21.0.

For manuscripts utilizing custom algorithms or software that are central to the research but not yet described in published literature, software must be made available to editors and reviewers. We strongly encourage code deposition in a community repository (e.g. GitHub). See the Nature Portfolio guidelines for submitting code & software for further information.

Data

Policy information about availability of data

All manuscripts must include a data availability statement. This statement should provide the following information, where applicable:

- Accession codes, unique identifiers, or web links for publicly available datasets
- A description of any restrictions on data availability
- For clinical datasets or third party data, please ensure that the statement adheres to our policy

Data and materials availability: All data needed to evaluate the conclusions in the paper are present in the paper and/or the Supplementary Materials.

Research involving human participants, their data, or biological material

Policy information about studies with human participants or human data. See also policy information about sex, gender (identity/presentation), and sexual orientation and race, ethnicity and racism.

Reporting on sex and gender	Not applicable.
Reporting on race, ethnicity, or other socially relevant groupings	Not applicable.
Population characteristics	Not applicable.
Recruitment	Not applicable.
Ethics oversight	Not applicable.

Note that full information on the approval of the study protocol must also be provided in the manuscript.

Field-specific reporting

Please select the one below that is the best fit for your research. If you are not sure, read the appropriate sections before making your selection.

☐ Life sciences ☐ Behavioural & social sciences ☒ Ecological, evolutionary & environmental sciences

For a reference copy of the document with all sections, see nature.com/documents/nr-reporting-summary-flat.pdf

Ecological, evolutionary & environmental sciences study design

All studies must disclose on these points even when the disclosure is negative.

Study description	In this study, we conducted a large-scale, three-year in-situ experiment of N and P addition based on the early soil formation environment in the retreating area of the Hailuogou Glacier. Our aim is to integrate additional soil processes, such as C process and nitrification process, into the theoretical framework of N-P coupling, beyond the processes of N fixation and P dissolution. Based on this approach, we seek to explore and reveal the positive feedback mechanisms among nutrient elements during the early stages of soil formation, providing empirical insights for the development of a unified theory of nitrogen-phosphorus coupling.
Research sample	The study location is situated within the most recently deglaciated region of the Hailuogou Glacier (coordinates: 29° 34' 08" N, 101° 59' 41" E). In May 2019, a substantial number of plots (>270) were established in the study area to conduct the in situ N and P addition experiments. The experiment included three treatments: N addition (+N), P addition (+P), and CK. We randomly allocated over 90 plots per treatment as replicates (i.e., the total number of plots assigned to the three treatments exceeded 270). This design also ensured the collection of 90 replicate samples per treatment (a total of 270 samples for the three treatments) by the end of the experiment.
Sampling strategy	The in-situ experiment was concluded in July 2022, and soil columns (0 - 5 cm) in each plot were collected as soil samples. The collection method was as follows: For each plot, the tubular polyethylene bag enclosing the intact soil core was excavated as a whole, and the soil at a depth of 0 - 5 cm of this core was collected and placed into a sterile sample bag as one soil sample. Altogether, we collected 270 fresh soil samples. Each of them was sieved through a 2-mm mesh before being divided into two subsamples. One subsample was stored at 4 °C for the measurement of soil physicochemical characteristics, and the other subsample was stored at -80 °C for genes analysis.
Data collection	According to the manufacturer's instructions, real-time quantitative polymerase chain reaction (qPCR) was conducted using a CFX96 optical real-time detection system (Bio-Rad, Hercules, CA, USA) to quantify the abundances of soil functional genes. The pH value of the soil was ascertained with a pH meter. Soil moisture content was quantified through the gravimetric method, involving the oven-drying of soil samples to a state of constant weight at a temperature of 105 °C. Soil temperature (ST) measurements were recorded at a depth of 10 cm using a thermometer at the site of sample collection. The bulk density (BD) of the soil was determined using a ring sampler (volume 100 cm3). The BD was then calculated as the ratio of the oven-dried soil weight to its known volume (100 cm3). The available P (AP) concentration in the soil was extracted using a 0.5 M NaHCO3 solution (pH 8.5) . TOC and TN in the soil were measured using an elemental analyzer (Elementar Vario EL). Soil DOC was measured by extracting with deionized water and using a total organic carbon analyzer (Elementar Vario TOC). The concentrations of ammonia N (NH_4^+-N) and nitrate N (NO_3^--N) were analyzed using a flow autoanalyzer (SEAL Analytical AutoAnalyzer 3) following extraction with 2 M KCl. Soil nitrogenase (Nase) activity was assessed by measuring the potential N fixation rate using the acetylene reduction assay. The total concentrations of LMWOAs were then measured by high-performance liquid chromatography (HPLC, Shimadzu, Japan) . Soil samples (0.5 g) were digested using an HCl–HF–HNO3–HClO4 mixture, and the concentrations of Fe, Al, and TP were determined using an inductively coupled plasma atomic emission spectrometer (ICP-AES, American Leeman Labs Profile). The collection procedure and record of these data were carried out by the authors (Hongyang Sun, Dong Yu, Jun Zhou, Yang Chen).
Timing and spatial scale	In May 2019, a substantial number of plots (>270) were established in the study area to conduct the in situ N and P addition experiments. Each plot measured 0.3 m × 0.3 m, with spacing between plots exceeding 4 m. The in-situ experiment was concluded in July 2022, and soil columns (0 - 5 cm) in each plot were collected as soil samples.

Data exclusions	The plots damaged by geological hazards(e.g., small-scale debris flows and landslides) were discarded.
Reproducibility	This experiment can be reproduced with the methods provided in the manuscript. This experiment design also ensured the collection of 90 replicate samples per treatment.
Randomization	In this study, a substantial number of plots (>270) were established in the study area to conduct the in situ N and P addition experiments. Each plot measured 0.3 m × 0.3 m, with spacing between plots exceeding 4 m. Given the complex microtopography, frequent geological disturbances (e.g., small-scale debris flows and landslides), and significant microenvironmental heterogeneity in the study area, to enhance the accuracy of experimental data and mitigate data loss from potential plot damage caused by natural disasters, we randomly allocated over 90 plots per treatment as replicates (i.e., the total number of plots assigned to the three treatments exceeded 270). This design also ensured the collection of 90 replicate samples per treatment (a total of 270 samples for the three treatments) by the end of the experiment.
Blinding	The random numbering process ensures that the sample order is independent across the testing, and that technicians have essentially no knowledge of the sample order or the relationships among the samples.

Did the study involve field work? ☒ Yes ☐ No

Field work, collection and transport

Field conditions	The field experiment was conducted within the most recently deglaciated region of the Hailuogou Glacier. The study site is at an elevation of 2990 meters above sea level. The annual precipitation at this location varies between 1586 and 2175 mm. The soil temperature, measured at a depth of 10 centimeters, fluctuates throughout the year from a minimum of -3 °C to a maximum of 21 ° C. The early soil within the study area has been exposed for a period not exceeding 18 months and is characterized by an absence of vegetation. The soil more closely resembles rock debris, notwithstanding the presence of a minor quantity of organic matter. The dominant P mineral in the study area is apatite, accounting for approximately 2% of the total soil mineral mass.
Location	The study location is situated within the most recently deglaciated region of the Hailuogou Glacier (coordinates: 29° 34' 08" N, 101° 59' 41" E)
Access & import/export	The field sites were full accessible for sampling and experiment research. All research complies to national and local laws and regulations.
Disturbance	A substantial number of plots (>270) were established in the study area to conduct the in situ N and P addition experiments. Each plot measured 0.3 m × 0.3 m, with spacing between plots exceeding 4 m. Given the complex microtopography, frequent geological disturbances (e.g., small-scale debris flows and landslides), and significant microenvironmental heterogeneity in the study area, to enhance the accuracy of experimental data and mitigate data loss from potential plot damage caused by natural disasters, we randomly allocated over 90 plots per treatment as replicates (i.e., the total number of plots assigned to the three treatments exceeded 270). This design also ensured the collection of 90 replicate samples per treatment (a total of 270 samples for the three treatments) by the end of the experiment. It ensured the robustness and reliability of our analysis and results.

Reporting for specific materials, systems and methods

We require information from authors about some types of materials, experimental systems and methods used in many studies. Here, indicate whether each material, system or method listed is relevant to your study. If you are not sure if a list item applies to your research, read the appropriate section before selecting a response.

Materials & experimental systems

n/a	Involved in the study	
☒	☐	Antibodies
☒	☐	Eukaryotic cell lines
☒	☐	Palaeontology and archaeology
☒	☐	Animals and other organisms
☒	☐	Clinical data
☒	☐	Dual use research of concern
☒	☐	Plants

Methods

n/a	Involved in the study	
☒	☐	ChIP-seq
☒	☐	Flow cytometry
☒	☐	MRI-based neuroimaging

Plants

Seed stocks	Not applicable.
Novel plant genotypes	Not applicable.
Authentication	Not applicable.
